# Diagnosis of spatial neglect and rehabilitation access for stroke survivors

**DOI:** 10.1080/28324897.2024.2375706

**Published:** 2024-07-11

**Authors:** Corey Morrow, Holden Gasque, Michelle Woodbury, Eyad Almallouhi, Annie Simpson, Kit Simpson

**Affiliations:** Division of Occupational Therapy, Medical University of South Carolina, Charleston, SC, USA

**Keywords:** Spatial neglect, occupational therapy, diagnosis, stroke, medical coding, I10

## Abstract

Spatial neglect in stroke survivors is associated with a decrease in quality of life. This disorder occurs in 20–80% of stroke survivors and up to 1/3 of stroke survivors will continue to experience chronic impairment. Occupational therapists are uniquely qualified to treat stroke survivors with spatial neglect due to their holistic approach but access to therapy is limited. Diagnostic coding is used to help determine appropriate reimbursement and continuation of care including rehabilitation services. The objectives of this study were to 1) identify the prevalence of diagnostic coding for spatial neglect in stroke survivors, and 2) identify the prevalence and types of rehabilitation for patients with diagnostic coding for spatial neglect. We completed a retrospective cohort analysis using 2018 and 2019 5% Medicare Limited Data Sets from the Centers for Medicare and Medicaid Services. We extracted all ischemic stroke survivors and stratified them by the presence of a secondary diagnostic code indicating spatial neglect. Rehabilitation Current Procedural Terminology codes were used to identify stroke survivors who received rehabilitation. Despite recommendations from clinical practice, only 4.9% had a diagnostic code for spatial neglect. Of those formally diagnosed, only 2.3% received outpatient occupational therapy after being discharged from acute care.

## 1. Introduction

Every year there are 795,000 new strokes in the United States (Virani et al., 2020). Spatial neglect is a disorder of lateralized spatial attention, characterized by the inability to attend, orient or respond to stimuli occurring in the space opposite the stroke lesion (Heilman & Rt, 1977) which can negatively impact activities of daily living (ADLs) such as dressing (Chen, Hreha, et al., 2015). This disorder occurs in 20–80% of stroke survivors (Chen et al., 2018; Esposito et al., 2021; Hammerbeck et al., 2019; Hreha et al., 2017) with both left and right cerebral hemisphere infarcts (Esposito et al., 2021). Stroke survivors with spatial neglect have longer hospital stays but poorer functional outcomes (Hammerbeck et al., 2019). Even after a typical inpatient rehabilitation stay, at least half of the stroke survivors with spatial neglect will continue to have persistent symptoms (Chen, Chen, et al., 2015), and up to one-third of stroke survivors will continue to experience chronic neglect lasting longer than a year (Karnath et al., 2011).

Spatial neglect is associated with a decrease in quality of life (Sobrinho et al., 2018), increased falls (Chen, Hreha, et al., 2015), and reduced balance (Nijboer et al., 2014). Additionally, anosognosia, or a poor insight to impairments (Nurmi Laihosalo & Jehkonen, 2014), causes treatment delays because stroke survivors aren’t aware of their unawareness and have difficulties learning compensatory or restorative techniques (Tham et al., 2001). Patients have described frustrations with having poor insight into their deficits (Tobler-Ammann et al., 2020), and a documented diagnosis of spatial neglect may allow for earlier and consistent patient/caregiver education.

Novel treatment strategies may improve spatial neglect (Barrett, 2021; Barrett & Houston, 2019), but access to stroke rehabilitation in the community is a significant barrier. In general, while 78% of stroke survivors receive rehabilitation during acute care (Freburger et al., 2018), only between 40% and 56% receive community-based rehabilitation (home health or outpatient therapy) (Prvu Bettger et al., 2015;). Outpatient occupational therapy is even more limited in rural and socially disadvantaged counties (Morrow et al., 2024).

While spatial neglect is common for stroke survivors, a lack of consistent terminology makes communication between clinicians and disciplines difficult and may contribute to reduced continuity of care (Chen et al., 2018; Chen, Zanca, et al., 2021). One study discovered 200 different terms used in research to refer to neglect or a subtype such as visual neglect, visuospatial neglect, visuo-spatial neglect, visual inattention, and visual-spatial inattention (Williams et al., 2021). Previous publications have described under-documentation for spatial neglect during inpatient rehabilitation. While occupational therapists, physicians, and nurses may document spatial neglect in clinical notes, this rarely translates to formal diagnostic coding (Chen et al., 2013).

Common coding systems are the International Classification of Diseases Version 10 (ICD-10) (World Health, 2004) for diagnoses and Current Procedural Terminology (CPT) codes for procedures such as rehabilitation services (Dotson, 2013). Diagnostic codes are used to standardize terminology , increase communication between disciplines, and help determine appropriate reimbursement (Hirsch et al., 2016) Codes are used by both primary and secondary users (ICD—ICD-10-CM—International Classification of Diseases, (ICD-10-CM/PCS Transition). Primary users include physicians, nurses, and medical coders who help apply the appropriate codes to patients and communicate with other clinicians. Secondary users use already documented codes to determine reimbursement, conduct health services research, and track the quality of care (Chang et al., 2016). Therefore, underreporting puts both user types at a disadvantage.

The **objectives** of this study were to 1) identify the prevalence of diagnostic coding for spatial neglect in stroke survivors, and 2) identify the prevalence and types of rehabilitation for patients with diagnostic coding for spatial neglect. To our knowledge, this is the first investigation into the prevalence of diagnostic coding of spatial neglect in Medicare stroke survivors and the rehabilitation they receive.

## 2. Methods

### 2.1. Dataset construction

We completed a retrospective cohort analysis using the Centers for Medicare and Medicaid Services (CMS) 2018 and 2019 5% Medicare Limited Data Sets (LDS). These data include demographic and billing information for a random sample of beneficiaries. Ischemic stroke survivors were identified using Diagnosis Related Group (DRG) codes. Patients were excluded if they died in acute care or were discharged to hospice as these patients are not candidates for rehabilitation. Additionally, patients had to be trackable through their Medicare benefits for 364 days past their initial stroke. Continuous variables are reported with means and standard deviations (SD) and categorical variables are reported with frequencies and percentages. Subsamples with less than 11 patients are included but specified as ‘n < 11’ to abide by CMS policy to protect patient confidentiality.

Stroke survivors with neglect were identified using ICD-10 codes R41.4 (Neurologic neglect syndrome) and I69.912 (Visuospatial deficit and spatial neglect following unspecified cerebrovascular disease) (World Health, 2004). Because there were so few stroke survivors diagnosed with neglect, we decided to combine all stroke survivors diagnosed with neglect into one group (Neglect Group).

There were two stroke severity variables used to describe the two groups. The National Institutes of Health Stroke Scale (NIHSS) was included; however, this is typically underreported in the LDS. Therefore, we also reported the Stroke Administrative Severity Index (SASI). This provides a level of stroke severity (0–31 points) based on the presence of ICD-10 codes for aphasia (4 points), coma (23 points), dysarthria/dysphagia (2 points), hemiplegia (6 points), neglect (5 points), need for nutritional infusion (6 points), and the need for tracheostomy and/or ventilation (10 points). Points are aggregated and categorized as Mild (0 points), Moderate (1–6 points), or Severe (7–31 points) (Simpson et al., 2018). Other descriptive variables included whether a stroke survivor received tissue plasminogen activator (tPA) or endovascular thrombectomy (eVT) during acute care, baseline comorbidities as described by the Charlson Co-Morbidity Index (D’Hoore et al., 1996), dual eligibility for Medicare and Medicaid (indicating lower-income patients), and discharge destinations after acute care.

Analyses were performed using SAS, version 9.4 (SAS Institute, Inc.; Cary, NC). The university’s Institutional Review Board deemed this non-human research and, therefore, did not require oversight.

### 2.2. Therapy evaluations

Outpatient and home health CPT codes (Appendix A) for OT and PT evaluations were extracted and consolidated into either ‘OT Evaluation’ or ‘PT Evaluation’. We did not include recertifications as our focus was on initial access of therapy not on continuation. The presence of CPT codes for therapy evaluations for PT/OT and home health/outpatient were compared for the Neglect Group and No Neglect Diagnosis Group.

### 2.3. Socially disadvantaged and rural subpopulations

We had two subpopulations of specific interest: rural and socially disadvantaged (SDA) stroke survivors. Rural and nonrural stroke survivors were identified using county Federal Information Processing System (FIPS) codes. SDA stroke survivors were identified using the Community Vulnerability Index (CVI). The CVI is weighted by factors such as poverty, income, education level, disability status, single-parent status, unemployment, housing, and transportation access (Registry, 2018). Stroke survivors residing in zip codes in the 90^th^ percentile for this index we considered SDA. Access to both outpatient and home health therapy were analyzed for these subpopulations.

## 3. Results

### 3.1. Demographics

Of the 9,076 stroke survivors in this dataset, only 4.9% were formally diagnosed with spatial neglect (Table 1). For those diagnosed with spatial neglect, the mean age was 77.5 (10.2), 56.1% were female, and 79.0% were White. For subpopulations, 13.8% of the Neglect Group lived in a rural setting and 7.9% lived in an SDA setting.

Stroke severity was higher in the Neglect Group versus the No Neglect Diagnosis Group in both the NIHSS (9.9 SD = 7.7 vs. 5.7 SD = 6.4) and SASI (8.9 SD = 5.3 versus 4.7 SD = 4.2). Length of stay (LOS), tPA, and eVT were also higher for the Neglect Group. Stroke survivors in the Neglect Group were discharged to skilled nursing (36.4%) or inpatient rehabilitation (31.5%) most frequently. For the No Neglect Diagnosis Group, the most frequent discharge destination was directly Home (31.9%).

### 3.2. Access to community-based therapy evaluations

Access to community-based therapy included evaluations for home health therapy, outpatient therapy, or no therapy (Table 2). In the Neglect Group, 56.3% of the patients received no home health or outpatient therapy upon discharge compared to 50.6% in the No Neglect Diagnosis Group. For those who received therapy in the Neglect group, 42.1% of the patients received home health physical therapy, 35.5% received home health occupational therapy, 35.5% received both home health physical therapy and occupational therapy and 57.9% received neither home health physical therapy nor occupational therapy.

As for outpatient therapy, access is very limited for both groups. For stroke survivors in the Neglect Group, 5.9% received outpatient physical therapy, 2.3% received outpatient occupational therapy, 2.0% received both outpatient physical therapy and occupational therapy. Almost 94% of the Neglect Group did not receive outpatient physical therapy or occupational therapy compared to 90.6% in the No Neglect Diagnosis Group.

### 3.3. Therapy access for subpopulations with neglect

For the Neglect Group who lived in rural communities, 31.2% of the received home health OT and 44.3% received home health PT (Table 3). For nonrural stroke survivors with neglect, 36.2% received home health OT and 41.7% received home health PT. For outpatient therapy,0% of the Neglect Group in rural communities received rehabilitation. However, 6.8% of the Neglect Group in nonrural communities received outpatient PT and 2.6% received outpatient OT.

For socially disadvantaged (SDA) communities, 65.7% of stroke survivors with neglect received no home health or outpatient therapy. This is compared to 55.5% living in non-SDA communities.

## 4. Discussion

### 4.1. Demographics

Only 4.9% of Medicare beneficiaries were formally diagnosed with an ICD-10 code having spatial neglect. This is dramatically less than a recent systematic review which reported the estimated prevalence of spatial neglect to be 30% (Esposito et al., 2021). While there are likely many more explanations, this may suggest either clinicians are not assessing for neglect, are not using the diagnostic code for neglect, or that this population did not have neglect.

Time, resources, and knowledge are all barriers to diagnosing spatial neglect (Chen, Zanca, et al., 2021). Therapists have expressed early discharges from inpatient rehabilitation specifically as a barrier to a more comprehensive evaluation which may include neglect (Chen, Hreha, et al., 2015; Chen, Zanca, et al., 2021). Spatial neglect diagnosis can be difficult and multiple assessments may need to be performed to identify the correct subtype of neglect (Grattan & Woodbury, 2017). Ironically, formally diagnosing neglect may lead to increased length of stays for inpatient rehabilitation. Diagnostic codes help determine levels of reimbursement and days approved for inpatient rehabilitation stays.

Clinicians have also expressed barriers to spatial neglect treatment as poor communication between disciplines and clinicians and a discontinuity throughout the continuum of rehabilitation care (Chen, Zanca, et al., 2021). Patients may not have self-awareness of their spatial neglect deficits (Ronchi et al., 2014), so clinician communication is particularly crucial as patients may not have the capacity to self-advocate for specialized treatment. Clinicians have previously identified the importance of formal documentation to help with this barrier (Chen, Zanca, et al., 2021).

Another important benefit of formal diagnostic coding is to aid in epidemiology, quality of care, and health services research (Chang et al., 2016; Chen, Zanca, et al., 2021). This is particularly timely as there is a push from the National Institutes of Health (NIH) for a rehabilitation-focused health services research (Frontera et al., 2017). Diagnostic coding also allows for the analysis of healthcare disparities to better understand the access to care for rural and SDA subpopulations (Meyer, 2011; Sanders et al., 2012).

### 4.2. Therapy evaluations

Despite ongoing treatment recommendations from clinical practice guidelines (Winstein Carolee et al., 2016), our results suggest that many stroke survivors with neglect might be discharged home without crucial rehabilitation services. Over 56% of stroke survivors with neglect were discharged into the community without home health or outpatient therapy. While some of these patients may have had a full recovery of their symptoms during acute or inpatient rehabilitation, it is likely these stroke survivors are still living with deficits that may impact quality of life (Karnath, 2015). Additionally, caregiver burden is high for stroke survivors with neglect that could be addressed with community-based rehabilitation (Chen et al., 2017). Treatment may even improve patients’ ability to return to work (Kerkhoff, 2021).

Spatial neglect experts typically approach treatment with multiple approaches due partially to the complex nature of the ailment (Chen et al., 2018). Outpatient therapy facilities may offer several specialized treatment options for spatial neglect including virtual reality (Morse et al., 2020; Ogourtsova et al., 2017), prism therapy (Chen, Diaz-Segarra, et al., 2021), and activity-based interventions (Liu et al., 2019). Unfortunately, as shown in our analysis, 93.9% of neglect patients do not receive outpatient therapy in the first year. Furthermore, no rural stroke survivors with neglect received outpatient therapy. This may be explained by a lack of therapists in rural areas (MacDowell et al., 2010). Recently, telerehabilitation has emerged as an option to improve access and specialty care for stroke survivors living in rural areas (Morse et al., 2020).

For stroke survivors certified by a physician as homebound, 60 days of home health rehabilitation is an option (Winstein Carolee et al., 2016). Though research specifically for home health treatments for spatial neglect is limited, stroke survivors may benefit in many ways. Spatial neglect rehabilitation in the patient’s home could help identify safety risks to reduce falls and help patients and caregivers adopt compensatory and restorative techniques for their ADL routines. Unfortunately, only 35.5% of those diagnosed with neglect received both home health occupational and physical therapy. Furthermore, 57.9% of stroke survivors with neglect received no home health and 65.7% of those living in SDA communities did not receive home health or outpatient therapy.

### 4.3. Future research needs

There is a need to better understand if the prevalence of neglect is accurately reflected in the diagnostic coding, and if it is not accurate, there is a need to identify the facilitators and barriers to improve coding for spatial neglect. Additionally, cost-effectiveness studies for spatial neglect treatments could help clinicians, insurers, and policymakers understand the importance of treatment. Finally, telerehabilitation is emerging as a possible option for spatial neglect (Morse et al., 2022). However, continued research on telerehabilitation efficacy and effectiveness is important, and understanding barriers to clinician and patient telerehabilitation use for spatial neglect will be imperative to promote widespread adoption.

### 4.4. Limitations

There were limitations to consider in our study. First, this was a retrospective study using only Medicare beneficiaries. Results may not be generalizable to other populations. Second, patients may have received therapy that was not billed to Medicare. Third, results may be affected by an under-ascertainment bias. Finally, this study only examined ischemic stroke survivors and the results may not be generalizable to transient ischemic attacks or hemorrhagic strokes.

## 5. Conclusions

Spatial neglect is likely underdiagnosed and underreported in acute, home health, and outpatient care. Increased use of diagnostic coding might potentially increase clinician awareness, increase inpatient rehabilitation length of stays and reimbursement, and lead to treatment. For stroke survivors who are diagnosed with neglect, community-based rehabilitation access is limited. This is particularly true for rural and socially disadvantaged populations.

## Figures and Tables

**Table 1.** Cohort characteristics.

Characteristics	Mean (SD) or N (%)	
	Neglect Group	No Neglect Diagnosis Group
	N = 442 (4.9%)	N = 8,634 (95.1%)
Age mean (SD) in years	76.5 (10.2)	76.2 (10.6)
<65 years old	43 (9.7%)	829 (9.6%)
65–75 years old	136 (30.8%)	2,768 (32.1%)
>75 years old	263 (59.5%)	5,037 (58.3%)
Female sex N (%)	248 (56.1%)	4,543 (52.6%)
Race N (%)		
White	208 (79.0%)	6,984 (80.9%)
Black	67 (15.2%)	1,096 (12.7%)
Hispanic	5 (1.1%)	119 (1.4%)
Other	21 (4.8%)	435 (5.0%)
Rural population N (%)	61 (13.8%)	1,159 (13.4%)
Socially Disadvantaged (SDA) population N (%)	35 (7.9%)	658 (7.6%)
Dual Eligibility Medicare/Medicaid N (%)	116 (26.2%)	1,800 (20.9%)
**Comorbidity/Severity**		
Charlson Comorbidity Index Mean (SD)	4.3 (2.2)	3.9 (2.2)
NIHSS Mean (SD)	9.9 (7.7)*	5.7 (6.4)**
SASI mean (SD)	8.9 (5.3)	4.7 (4.2)
SASI Categories:		
Mild N (%)	42 (9.5%)	2,850 (33.0%)
Moderate N (%)	138 (31.2%)	3,611 (41.8%)
Severe N (%)	262 (59.3%)	2,173 (25.2%)
Received tPA (%)	54 (12.2%)	747 (8.7%)
Received eVT (%)	30 (6.8%)	215 (2.5%)
Hospital LOS Mean (SD) days	6.6 (4.8)	5.0 (3.8)
**Hospital Discharge Destination**		
Home N (%)	69 (15.6%)	2,755 (31.9%)
Home with Home Health N (%)	47 (10.6%)	1,234 (14.3%)
Inpatient Rehabilitation N (%)	139 (31.5%)	2,033 (23.6%)
Skilled Nursing Facility N (%)	161 (36.4%)	2,156 (25.0%)
Transferred N (%)	15 (3.4%)	287 (3.3%)
Other N (%)	11 (2.5%)	169 (2.0%)

SASI: Stroke Administrative Severity Index; tPA: tissue plasminogen activator; eVT: endovascular thrombectomy; LOS: length of stay.

* N = 218;

** N = 3,685.

**Table 2.** Access to community-based rehabilitation evaluations.

Therapy Type	N (%)	
	Neglect Group	No Neglect Diagnosis Group
	(N = 442)	(N = 8,634)
Home health therapy		
Physical therapy	186 (42.1%)	3,817 (44.2%)
Occupational therapy	157 (35.5%)	2,965 (34.3%)
Both physical/occupational therapy	157 (35.5%)	2,897 (33.6%)
Neither physical/occupational therapy	256 (57.9%)	4,749 (55.0%)
Outpatient therapy		
Physical therapy	26 (5.9%)	761 (8.8%)
Occupational therapy	<11 (2.3%)	167 (1.9%)
Both physical/occupational therapy	<11 (2.0%)	119 (1.4%)
Neither physical/occupational therapy	415 (93.9%)	7,825 (90.6%)
No home health or outpatient therapy	249 (56.3%)	4,330 (50.6%)

**Table 3.** Therapy access for subpopulations with neglect.

Therapy Type		Neglect Group N (%)		
	Rural	Nonrural	SDA	Non-SDA
	(13.8%)	(86.2%)	(7.9%)	(92.1%)
Home health therapy				
Occupational therapy	19 (31.2%)	138 (36.2%)	9 (25.7%)	148 (36.4%)
Physical therapy	27 (44.3%)	159 (41.7%)	<11 (28.6%)	176 (43.2%)
Outpatient therapy				
Physical therapy	0 (0.0%)	26 (6.8%)	<11 (7.7%)	24 (5.9%)
Occupational therapy	0 (0.0%)	<11 (2.6%)	<11 (6.25%)	<11 (1.7%)
No home health or outpatient therapy	34 (55.7%)	215 (56.4%)	23 (65.7%)	226 (55.5%)

## Appendix

**Appendix A.** Rehabilitation billing codes

Code	Description
97163	Physical Therapy Evaluation High Complexity
97162	Physical Therapy Evaluation Moderate Complexity
97161	Physical Therapy Evaluation Low Complexity
G0151	Home Health Physical Therapy
97167	Occupational Therapy Evaluation High Complexity
97166	Occupational Therapy Evaluation Moderate Complexity
97165	Occupational Therapy Evaluation Low Complexity
G0152	Home Health Occupational Therapy
